# Supplementary figures and images for: The genome and phenome of the green alga Chloroidium sp. UTEX 3007 reveal adaptive traits for desert acclimatization
Source: eLife. 2017 Jun 17;6:e25783. doi: 10.7554/eLife.25783 (PMC5509433; doi:10.7554/eLife.25783)

PM01 (Carbon Sources)

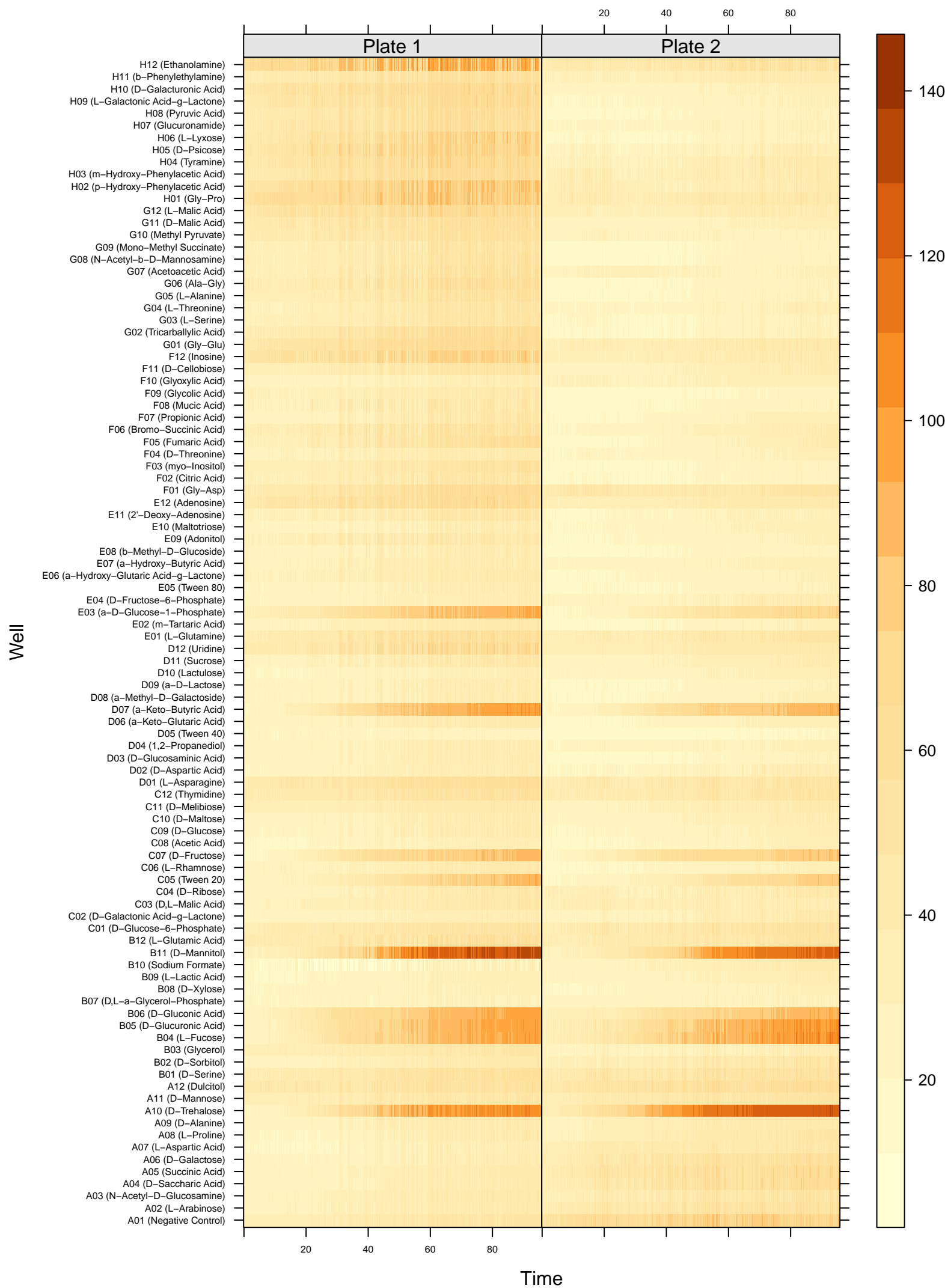

Supplement: Figure 4—source data 1. — DOI: http://dx.doi.org/10.7554/eLife.25783.015 [file elife-25783-fig4-data1.zip › level plot PM01.pdf]

PM02 (Carbon Sources)

Well

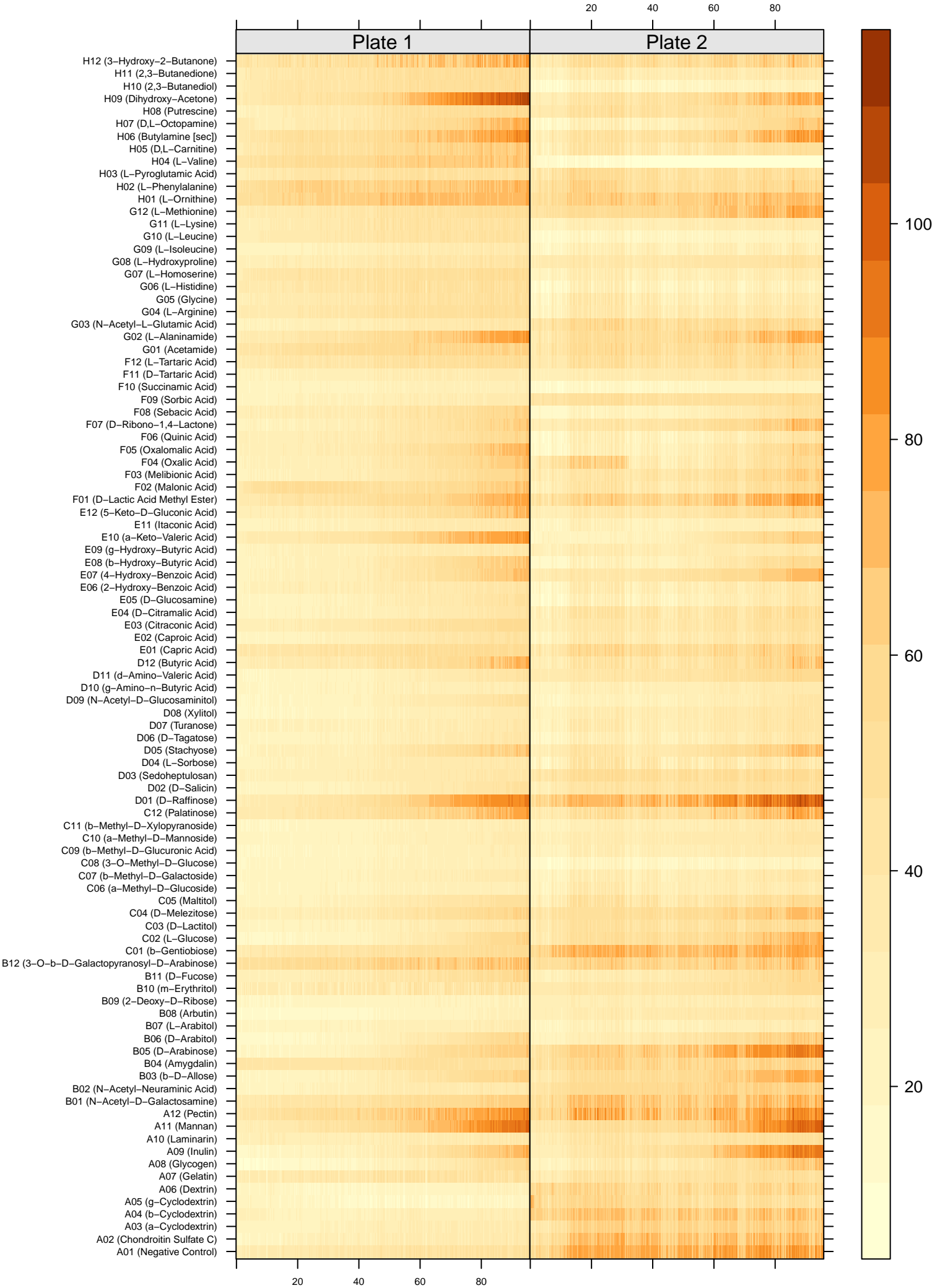

Time

Supplement: Figure 4—source data 1. — DOI: http://dx.doi.org/10.7554/eLife.25783.015 [file elife-25783-fig4-data1.zip › level plot PM02.pdf]

# PM03 (Nitrogen Sources)

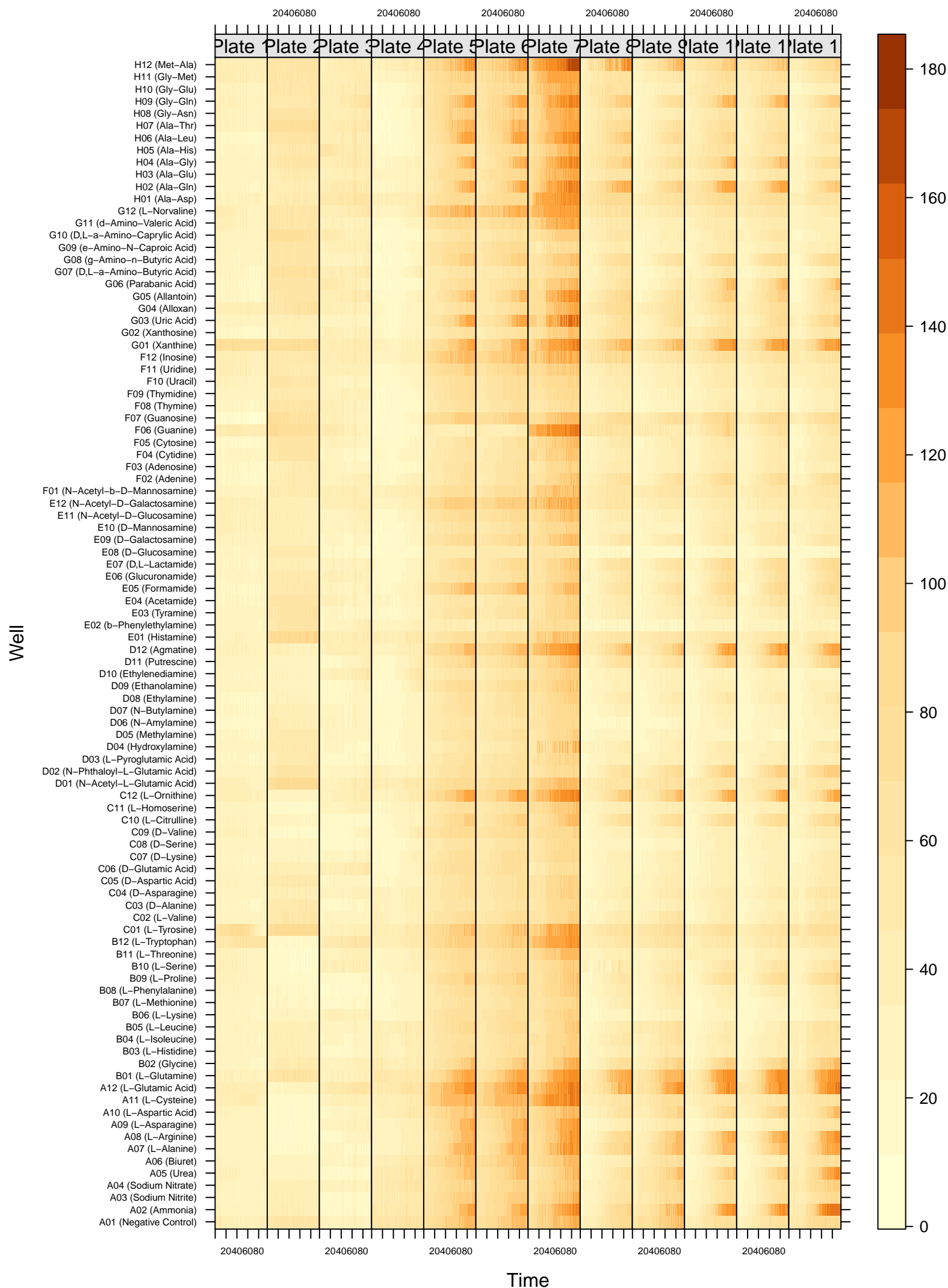

Supplement: Figure 4—source data 1. — DOI: http://dx.doi.org/10.7554/eLife.25783.015 [file elife-25783-fig4-data1.zip › level plot PM03.pdf]
